# GelMA/PEGDA microneedles patch loaded with HUVECs-derived exosomes and Tazarotene promote diabetic wound healing

**DOI:** 10.1186/s12951-022-01354-4

**Published:** 2022-03-19

**Authors:** Meng Yuan, Kun Liu, Tao Jiang, Shengbo Li, Jing Chen, Zihan Wu, Wenqing Li, Rongzhi Tan, Wenying Wei, Xiaofan Yang, Honglian Dai, Zhenbing Chen

**Affiliations:** 1 Department of Hand Surgery, Union Hospital, Tongji Medical College, Huazhong University of Science and Technology, Wuhan, 430022 China; 2 State Key Laboratory of Advanced Technology for Materials Synthesis and Processing, Wuhan University of Technology, Wuhan, 430070 China; 3 Department of Hand and Foot Surgery, Huazhong University of Science and Technology Union Shenzhen Hospital, Shenzhen, 518052 China

**Keywords:** Microneedle, GelMA hydrogel, Exosomes delivery, Drug release, Diabetic wound healing

## Abstract

Clinical work and research on diabetic wound repair remain challenging globally. Although various conventional wound dressings have been continuously developed, the efficacy is unsatisfactory. The effect of drug delivery is limited by the depth of penetration. The sustained release of biomolecules from biological wound dressings is a promising treatment approach to wound healing. An assortment of cell-derived exosomes (exos) have been proved to be instrumental in tissue regeneration, and researchers are dedicated to developing biomolecules carriers with unique properties. Herein, we reported a methacrylate gelatin (GelMA) microneedles (MNs) patch to achieve transdermal and controlled release of exos and tazarotene. Our MNs patch comprising GelMA/PEGDA hydrogel has distinctive biological features that maintain the biological activity of exos and drugs in vitro. Additionally, its unique physical structure prevents it from being tightly attached to the skin of the wound, it promotes cell migration, angiogenesis by slowly releasing exos and tazarotene in the deep layer of the skin. The full-thickness cutaneous wound on a diabetic mouse model was carried out to demonstrate the therapeutic effects of GelMA/PEGDA@T + exos MNs patch. As a result, our GelMA/PEGDA@T + exos MNs patch presents a potentially valuable method for repairing diabetic wound in clinical applications.

**Graphic Abstract:**

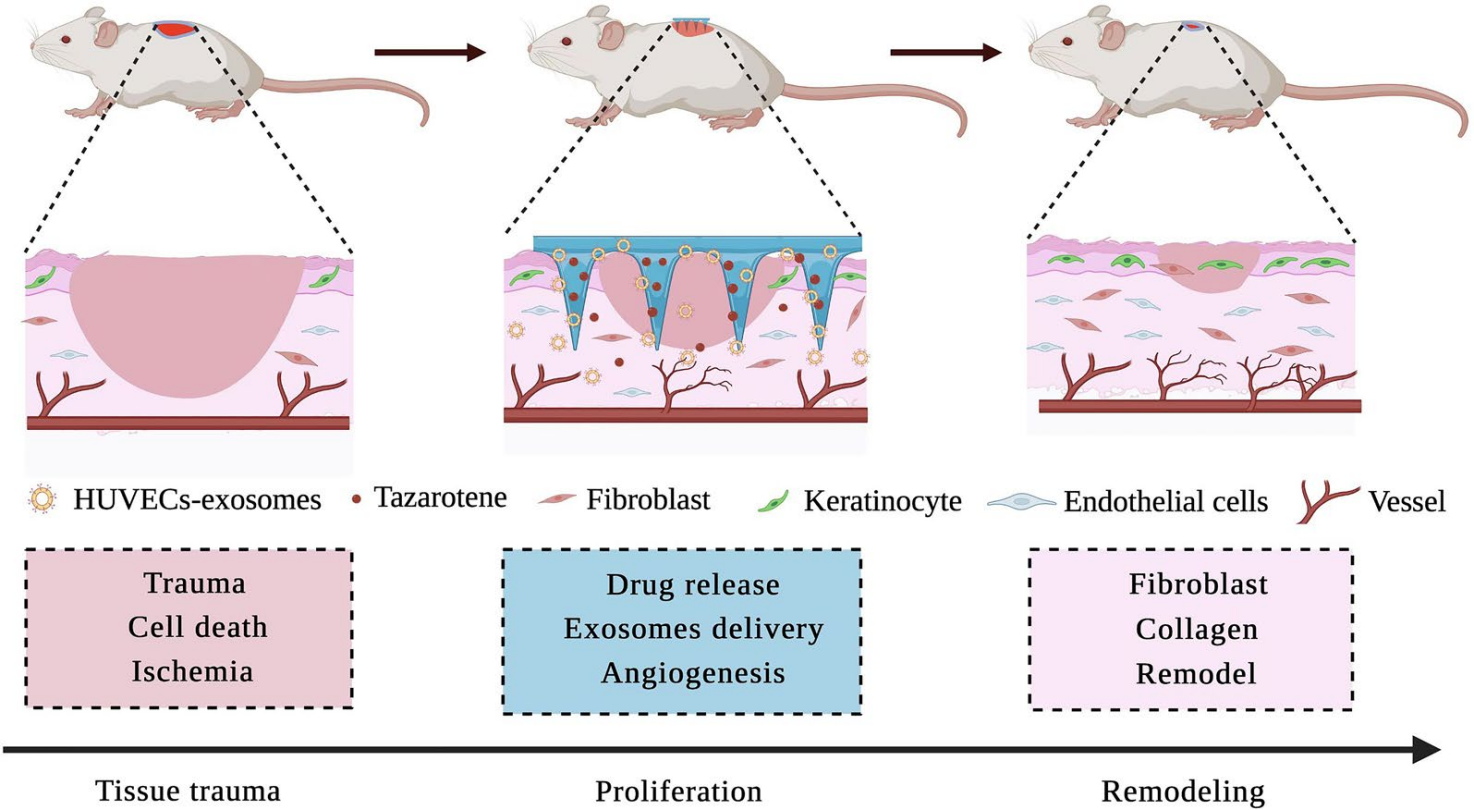

**Supplementary Information:**

The online version contains supplementary material available at 10.1186/s12951-022-01354-4.

## Introduction

The global prevalence of adult diabetes is projected to increase to 7.7% by 2030 [1], complications caused by diabetes seriously threaten the health of patients, resulting in decreased quality of life, increased medical costs and mortality [2–4]. Hyperglycemic stimulation is one of the factors that cause vascular injuries including diabetic foot ulcers (DFU) [5]. The treatment of DFU consists primarily of debridement, anti-infection and wound dressing, however, none of these provides satisfactory therapeutic effects [6]. It is estimated that approximately 60% of amputations are caused by DFU, which is the leading cause of hospitalization in patients with diabetes [7, 8]. Therefore, designing ideal wound healing materials would require meeting the needs of complete wound healing, which is of paramount importance.

The traditional wound dressing procedure is lengthy and a source of a considerable psychological burden on patients. At present, the diabetic wound healing treatment methods primarily include local drug administration and cellular therapy [9]. Biomaterials with controlled-release of biomolecules are promising materials for diabetic wound repair and should, therefore, be considered. Biomedical dressings that can efficiently deliver drugs/cells have been studied extensively in wound healing therapy and skin regeneration [10–12]. The ideal biological dressing scaffold stent delivery system must ensure the long-term release of biomolecules [13].

As a minimally invasive tool, microneedles (MNs) have attracted progressively more attention in recent years. They have many outstanding merit including painlessness, self-management and easy handling. Numerous studies have shown that MNs can successfully deliver small molecule drugs and macromolecular drugs (such as insulin, vaccines, proteins and chemotherapy drugs) through the skin. MNs also have the advantage over traditional bandages and hydrogels of transporting drugs through deep skin and improving drug delivery efficiency. MNs are divided into soluble MNs and insoluble MNs. Insoluble MNs are more conducive to maintaining stability and sustaining the release of drugs than soluble MNs. Materials commonly used in MNs preparation include gelatin, PLGA, PVA and chitosan [14–17]. Previous investigations and applications have commonly used subcutaneous injections, spraying, and oral delivery systems for drug delivery. However, these methods either bring pain to patients (subcutaneous injection) or result in massive drug waste (spraying method) without achieving the desired effect. Researchers have made extraordinary efforts to explore the feasibility of biomolecules release for wound repair. For example, surgical dressings, hydrogels and recently developed MNs have achieved remarkable results. However, unlike surgical dressings and traditional hydrogels, MNs ensure better subcutaneous injection better through percutaneous tissue, and render more effective drug release to the wound without apparent irritation.

Exosomes (exos) are cell-released small vesicles with a diameter of 50–150 nm, they regulate communication between cells by transferring contained bioactive cargos including miRNAs, mRNAs and proteins [18–20]. Recent studies have widely scrutinized the effects of exos from different sources on wound healing [21–23]. Different mesenchymal stem cells (MSCs) reportedly promote wound healing, platelet-rich plasma, fibrocyte, and macrophage-derived exos allegedly facilitate diabetic skin regeneration. Angiogenesis is fundamental to tissue growth, with inappropriate angiogenesis resulting in abnormal wound healing [24]. Endothelial cells participate in angiogenesis, however, endothelial cells-derived exos have rarely been studied in diabetic wound healing. Tazarotene is retinoic acid kind of drug that effectively promotes angiogenesis, hair follicle and collagen regeneration in wound repair. However, its poor water solubility and low transdermal efficiency limit its application [25–28]. Therefore, a loading method that combines tazarotene with exos to treat diabetic wounds is needed.

Based on this premise, we sought to encapsulate the exos and tazarotene in MNs to avoid the loss of the biomolecules and injecting them into the wound site to increase the use effect. We used GelMA and PEGDA to prepare MNs, compounded exos in MNs, and grafted β-CD-AOI_2_ to carry tazarotene. MNs promote the directional release of drugs and exos in a mouse model, and accelerate cell proliferation, migration and angiogenesis both in vitro and in vivo. We also hypothesized that endothelial cells-derived exos contribute to accelerate the diabetic wound repair.

## Methods and materials

### GelMA preparation

GelMA was synthesized according to a previously described protocol [29]. Briefly, 10 g of gelatin (Sigma-Aldrich) was added to phosphate-buffered saline (PBS) to obtain 10wt% solution under the 50 ℃. Then, 8 ml of methacrylic anhydride (Aladdin, Shanghai, China) was steadily added to the solution with stirring for 6 h at 50 °C. Finally, 500 ml of warm PBS was added to end the reaction. Dialysis using a 13,000 molecular weight dialysis bag for 3 days to remove unreacted small molecules and freeze-dried to collect the product for future use.

### β-CD-AOI_2_ preparation

β-CD-AOI_2_ was synthesized according to a previously described protocol [30]. A simple nucleophilic reaction of ethyl isocyanate acrylate (AOI) (Aladdin, Shanghai, China) with cyclodextrin (Sinopharm Chemical Reagent Beijing Co. Ltd, China) gave cyclodextrin with double bond. 5 g cyclodextrin was dissolved in 50 ml DMF (Sinopharm Chemical Reagent Beijing Co. Ltd, China) containing 20 μl tin (II) 2-ethylhexanoate (Aladdin, Shanghai, China), and 3 ml AOI was slowly added dropwise to the solution to react for 6 h. The whole system was carried out in N_2_ atmosphere. The reaction product was recrystallized with acetone and dried under vacuum to obtain the final product for subsequent experiments.

### Characterization of hydrogel

The 1H NMR spectra of the GelMA and β-CD-AOI_2_ were collected from 0.7 ml sample (10 mg ml^−1^) dissolved in D_2_O by NMR system (Bruker AVIII500 M, Switzerland). In addition, in order to characterize the structural characteristics of PEGDA (Aladdin, Shanghai, China) and GelMA, Fourier transform infrared spectroscopy (FT-IR) (Therno Nicolet, Nexus/Nexus, USA) was used. FT-IR spectra were collected in transmission mode from 4000 cm^−1^ to 400 cm^−1^.

The mechanical properties of hydrogels with different PEGDA contents (0, 0.5%, 1%, 1.5%, 2%) were analyzed by a low-force mechanical testing system. Hydrogels with a diameter of 1 cm and a height of 4 mm were prepared for testing mechanical properties. The maximum loading force was set to 50.0 N, and the downward speed of the force sensor was set to 1 mm/min. The variation curve of compression force with deformation is recorded. The microstructure of hydrogels was analyzed by scanning electron microscopy. Firstly, the normal hydrogel was brittlely broken after freeze-drying, and its cross-section was observed and analyzed by scanning electron microscopy (SEM, JSM-4800, Japan Hitachi). At the same time, we concentrated the precursor solution in the vacuum environment, and then obtained the hydrogel by photo crosslinking, and finally carried out a similar SEM analysis.

### Preparation and characterization of GelMA/PEGDA MNs

The MNs were fabricated according to a vacuum method. Briefly, 1 g of GelMA was dissolved in 10 ml of PBS solution at 50 °C until fully dissolved. 25 mg Photoinitiator lithium acylphosphinate salt (LAP 0.05%, g/ml) were added to the GelMA solution for MNs fabrication. 100 μl of the above solution was poured over a polydimethylsiloxane (PDMS) mold containing an array of MNs cavities. The MN was placed in a vacuum environment at 50 °C remove air bubbles at the bottom of the MN mold while maintaining the dissolved state of GelMA. After centrifugation, the solution was exposed to UV light for gel. Finally, the MN arrays were detached from the PDMS mold after keeping the mold in the dark for 24 h to dry the GelMA/PEGDA MNs at room temperature.

To prepare a GelMA/PEGDA MN loaded with tazarotene (Shanghai, China) and exos, an appropriate amount of tazarotene (1 mg/10 ml) was dissolved in a precursor solution containing PEGDA, β-CD-AOI_2_ and GelMA. Then, after the dissolution of tazarotene was complete, the exos (100ug/ml) were added and mixed evenly, and 500 ul of matrix solution was filled into PDMS mold for MN preparation.

The prepared MNs were put into 50 ml PBS solution for degradation experiment. The MNs were taken out at different time points, wiped with filter paper, and dried after freezing. The MNs were characterized by scanning electron microscope (SEM, JSM-4800, Hitachi, Japan).

The drug release test was further carried out. Each MN sample was immersed in 10 ml PBS (pH = 7.4) at 37 °C. At each determined time point (1 d, 2 d, 3 d, 4 d, 5 d, 6 d, 7 d), 2 ml of release medium was added into 2 ml of methanol, and then ultrafiltrated for detection. The release medium extracted from the original release system was replaced with fresh PBS of the same volume. The amount of tazarotene released from the mixture was analyzed by microplate reader at 351 nm. In vitro, a GelMA/PEGDA@Tazarotene + exosomes (GelMA/PEGDA@T + exos) MNs patch was immersed with PBS at 37 °C. The number of exos proteins were detected using Micro BCA Protein Assay Kit (Thermo, 23,235, China) following the manufacturer’s instructions. In vivo, MNs patch loaded with PKH26 labeled HUVECs-exos and tazarotene was covered to the shaved back skin of the diabetic C57BL mice, the skin covered with MNs patch was harvested and fluorescence detection was then performed on day 2, the original MNs patch was continued to be covered to the other area of the skin, the fluorescence detection was then performed on day 4, and so on, the same treatment was carried out on day 6 and 8.

### Cell isolation and culture

Human immortalized keratinocytes (HaCAT) (#GDC106, CCTCC) and human umbilical vein endothelial cells (HUVECs) (#GDC166, CCTCC) were obtained from the China Center for Type Culture Collection (CCTCC, Wuhan, China) and cultured following the manufacturer’s instructions. HaCAT and HUVECs were cultured in Dulbecco's modified Eagle Medium (DMEM) containing 10% fetal bovine serum (FBS). Cells were cultured in a humidified atmosphere containing 5% CO_2_ at 37 °C. Human foreskin fibroblasts were isolated using previously described protocols [31].

### HUVECs-exos isolation and identification

Exosomes were isolated using a previous protocol [32]. When HUVECs reached 85–90% confluence, they were grown in an exos-free culture medium. The FBS (Serapro) used for culturing HUVECs was ultracentrifugated at 120,000 g overnight to remove contained extracellular exos. The culture medium was obtained and centrifuged at 1,000 g for 10 min to remove dead cells and 4,000 g for 25 min to eliminate cell debris. The supernatants were then moved to Amicon®Ultra-15 Centrifugal Filter (Millipore), and centrifuged at 13,000 g for 1 h. Next, the supernatants were centrifuged at 120,000 g for 80 min and 130,000 g for 70 min at 4 °C to obtain exos. The exos were washed once with PBS to remove contaminating proteins and stored at −80 °C for the next experiences. The pellets were resuspended in PBS and stored at −80 °C.

The qualification of HUVECs-exos was performed by transmission electron microscope (SEM, JSM-4800, Hitachi, Japan). The size distribution of HUVECs‐exos was measured by Nanoparticle tracking analysis (NTA; Beckman Coulter). The total protein level was quantified Pierce BCA Protein Assay Kit (Aspen, China).

### HUVECs-exos labeling and internalization assay

To determine uptake of the HUVECs-exos by HaCAT, fibroblasts and HUVECs, HUVECs-exos were incubated with red fluorescent dye (PKH26, Sigma, USA) for 5 min and the neutralize redundant dye was removed by 0.5% BSA/PBS. Then, the residual dye was removed by centrifugation again to obtain the labeled HUVECs-exos. Cells were seeded in the confocal dish, and then treated with different concentrations (0, 5, 10, 20 ug/ml) of labeled HUVECs-exos. The cells were washed with PBS after incubation for 24 h, and then fixed with paraformaldehyde (4%) for 10 min. FITC phalloidin (Yeasen Biotech Co., Shanghai, China) and DAPI (Solarbio, Beijing, China) were used to stain the cytoskeleton and nucleic respectively.

### Biocompatibility of the GelMA/PEGDA hydrogel in vitro

HaCAT, HUVECs, and Human foreskin fibroblasts were used to investigate the toxicity of the GelMA/PEGDA hydrogel in vitro. GelMA/PEGDA hydrogel was immersed in a complete medium containing DMEM (high glucose) with 10% FBS for different periods of time (0 h, 24 h, 48 h, 72 h). Then the extract liquid was collected for the biocompatibility testing. The LIVE/DEAD assay (Beyotime, Shanghai, China) was used to test the cell activity.

### CCK-8 assay

Cell viability was estimated by the CCK-8 assay (Dojindo, Shanghai, China). Approximately 1.2 × 10^4^ cells were seeded in 96-well plates with 100 μl extract liquid per well for 24 h, with three parallel controls for each group. Next, 10 μl of the working reagent was added to each well, and incubated at 37 °C for 2 h.

### Scratch assay

The scratch assay was performed in a 12-well plate. When the cells reached approximately 95% confluency with the extract liquid (48 h) per well, the scratch was made by using a 200 μl pipette tip. A phase contrast microscope was used to visualize the images. The images were analyzed with Image J version 5.0.

### In vitro tube formation assay

HUVECs (2.5 × 10^4^ cells per well) were seeded with the extract liquid (48 h) per well in 96-well culture plates that had been coated with 70 μl Matrigel Basement Membrane Matrix (BD Biosciences, CA, USA). Tube formation was detected by using the microscope at 0 h, 2 h, and 6 h incubation. Results are represented as mean ± SEM in three in- dependent experiments.

### Diabetic wound healing model

All animal experimental procedures were approved by the Institutional Animal Care and Use Committee of Tongji Medical College, Huazhong University of Science and Technology. Eighteen healthy eight-week-old male C57BL mice, weighing 20–23 g, were purchased from the Experimental Animal Center, Tongji Medical College, Huazhong University of Science and Technology. Streptozotocin (STZ) was used to induce healthy C57BL mice to develop diabetes by intraperitoneal injection. The blood glucose levels higher than 16.7 mmoll^−1^ at least 1 month were defined as diabetic mice model. Then the mice randomized into four groups randomly (n = 7 per group): the control group, GelMA/PEGDA MNs patch group, GelMA/PEGDA@Tazarotene (GelMA/PEGDA@T) MNs patch group, GelMA/PEGDA@Tazarotene + exosomes (GelMA/PEGDA@T + exos) MNs patch group. The C57BL mice were anesthetized by 0.3% phenobarbital sodium (0.1 ml/10 g) through intraperitoneal injection. A 10-mm full-thickness cutaneous wound was excised on the midline line of the mouse back. Silicone ring was sutured to the wound edge to prevent wound contraction. The wounds were covered with PBS, GelMA/PEGDA MNs patch, GelMA/PEGDA@T MNs patch and GelMA/PEGDA@T + exos MNs patch. Digital photographs were taken at days 0, 5, 10 and 15. The wound area was measured using the Image J software.

### Histology and immunofluorescence staining

After 15 days, the skin was cut into 10 mm*12 mm strips. Under the mechanical testing machine, the 50 N sensor stretched the skin at a speed of 1 mm/min. The wound area skin was removed and fixed with 4% paraformaldehyde for 48 h. The samples were embedded in paraffin after dehydration. A microtome was used to obtain 5 μm-thick serial sections, which were stained with hematoxylin–eosin (H&E), Masson trichrome, and immunofluorescence analysis. The sections of wound areas were respectively incubated with primary antibodies α-smooth muscle actin (Abcam, 1:200) and CD31 (Abcam, 1:200) overnight at 4 °C. Then they were incubated with the second antibody (Servicebio) for 1 h at room temperature. Images were taken by using a fluorescence microscope and then analyzed by Image J software.

### Statistical analysis

Statistical analysis of data was performed by the unpaired Student's t-test and one-way ANOVA among multiple groups. P < 0.05 was considered significant. Statistical analysis was performed with the GraphPad Prism v 5.0 software. Data represent the mean ± SD of three independent experiments.

## Results

### Preparation and properties of hydrogels

**Fig. 1:**
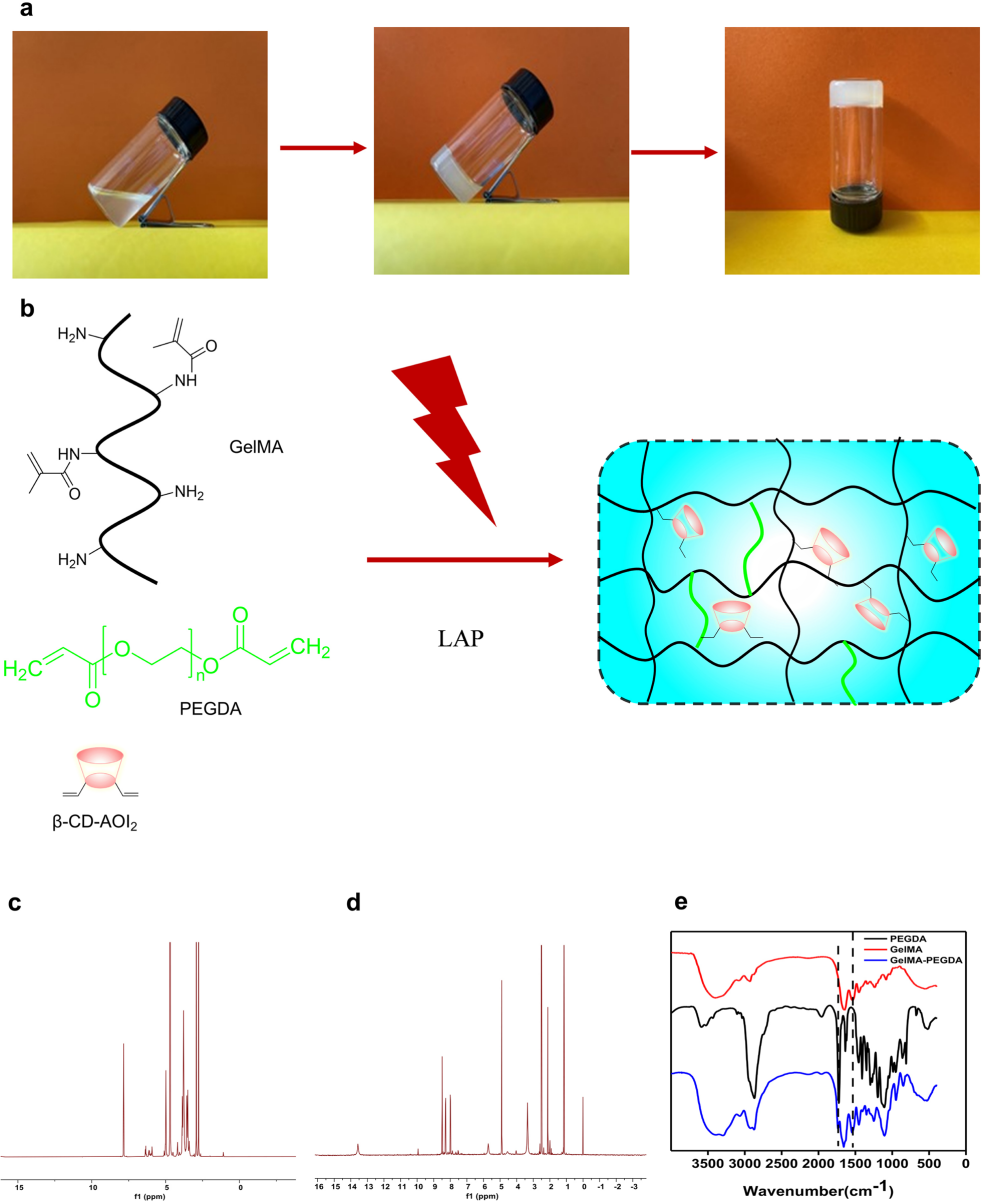
Morphologies of GelMA hydrogels. **a** Formation Process of Hydrogels. **b** Formation Mechanism of Hydrogels. **c** 1H NMR spectroscopy of β-CD-AOI_2_. **d** 1H NMR spectroscopy of GelMA. **e** The FTIR of PEGDA, GelMA, and GelMA-PEGDA

Our MNs patch design aims to release targeted drugs and exos for diabetic wound repair. To obtain stable MNs, we used light-curable gelatin as the primary material, and simultaneously introducing cyclodextrin, which reacts with double bonds to realize drug loading. The addition of short chain PEGDA was used to enhance the cross-linked network. The mechanism of hydrogel formation is shown in Fig. 1a, b.

The NMR of gelatin and cyclodextrin were obtained (Fig. 1c, d), the results from the integral analysis of the 1H NMR peak and matrix assisted laser desorption/ionization time-of-flight mass spectrum (MALDI-TOF–MS) of β-CD-AOI_2_ indicated that approximately two-CH=CH_2_ bonds were grafted at each β-CD-AOI_2_ molecule. The infrared results showed that the vibration peak of the amide in gelatin was at 1539 cm^−1^, and the stretching vibration peak of the C = O in amide was at 1649 cm^−1^, which existed in the infrared spectra of gelatin and the infrared spectra of the reactants (Fig. 1e). The stretching vibration peak of the C=O in the carbonate was at 1754 cm^−1^, which existed in the neutralization products of PEGDA, indicating that the products contained PEGDA and gelatin.

The drug loaded cyclodextrin can be connected with gelatin or PEGDA via double bonds, enabling effective drug loading. At the same time, the synthesized GelMA and PEGDA can be photocrosslinked with photoinitiator LAP under UV light [33]. The plasticity of the whole solution makes it more convenient for MNs preparation. we evaluated the impact of the PEGDA content on the properties of hydrogels to determine the optimal mechanical strength of hydrogels for MNs preparation. Notably, the compressive strength of MNs crucially depends on the concentration of PEGDA to maintain the penetration of MNs, the addition of PEGDA amplified the strength of hydrogels, however, excessive PEGDA rendered hydrogels brittle (Additional file 1: Fig. S1). Because PEGDA is a short chain, an appropriate addition can increase the cross-linking network, but introducing of too many short chains could also reduce the gel toughness. Concentrating the mixed precursor solution in a vacuum environment for a specific amount of time saw the solution density increase and the molecular chains become denser, with the microstructures in Additional file 1: Fig. S2g–l significantly denser than those in Additional file 1: Fig. S2a–f, the pore size of the hydrogel markedly decreased after concentration. Therefore, the concentrated solution was also conducive to preparing MNs patches.

### Morphologies and degradation performance of GelMA/PEGDA MNs

**Fig. 2:**
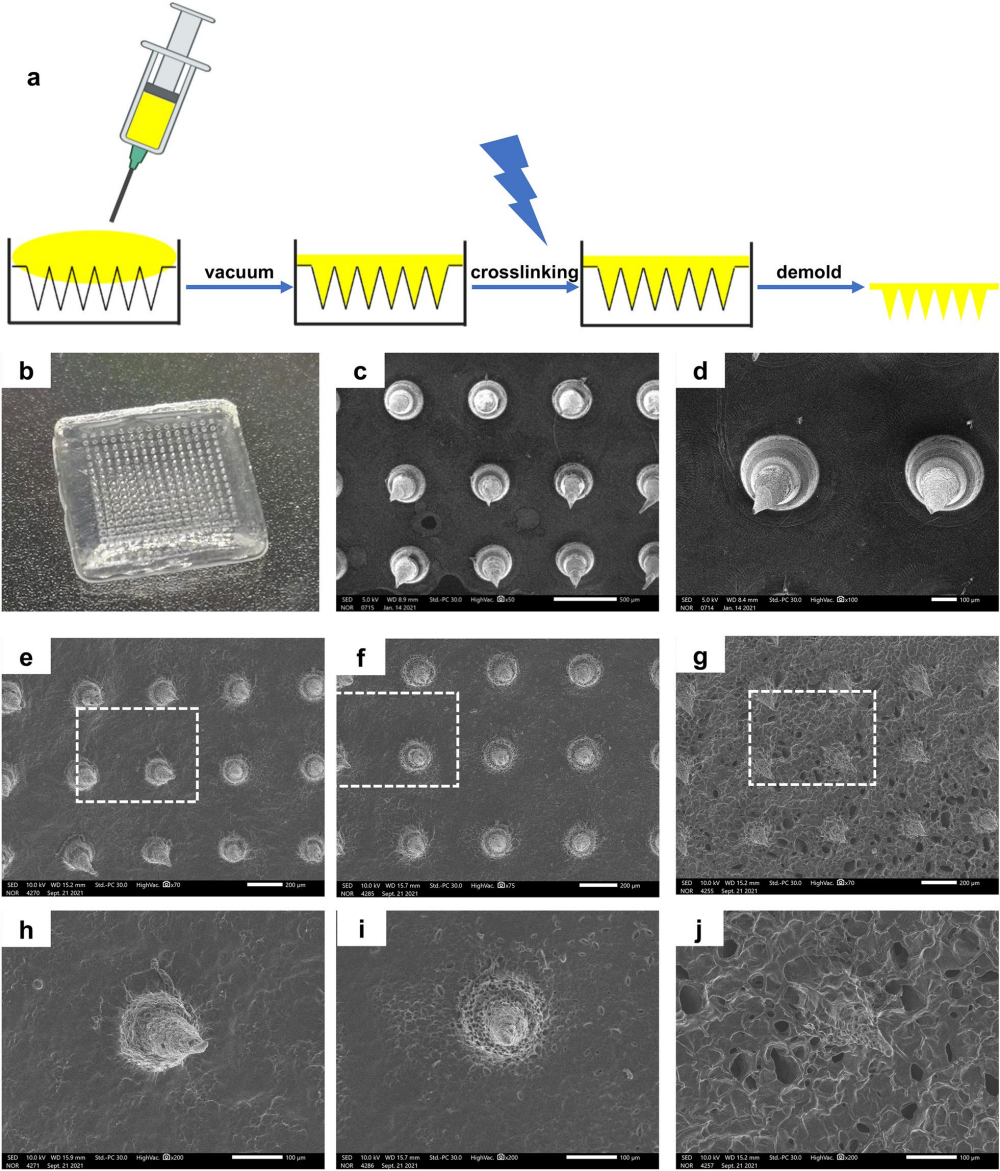
Morphology and degradation of GelMA/PEGDA MNs. **a** Preparation process GelMA MNs. **b** Physical picture of MNs patch. **c**, **d** SEM images of MNs. Scale bar in **C**: 500 μm, scale bar in **D**: 100 μm. **e**–**g** Degradation experiment of MNs (Day 1, Day 3, Day 5), scale bar: 200 μm. **h**–**j** Partial enlarged view of white box of scale bar: 100 μm

In a typical experiment, GelMA/PEGDA MNs were conducted by micromolding approach [34, 35]. Briefly, a predetermined amount of GelMA and PEGDA pre-gel solution was loaded into the MN mold, and kept the mixture in a vacuum environment at 50 ℃ for 30 min and maintained the atmosphere at a specific humidity to prevent excessive loss of moisture by the hydrogels. Subsequently, the mold filled with GelMA and PEGDA pre-gel solution was solidified in a low temperature environment, and the bubbles up the solution were easily removed. In the end, it was peeled off as an integral after UV irradiation. The same process was employed in the preparation of MNs that use β-CD-AOI_2_ to load drugs. Finally, a 16*16 array was obtained, and PEGDA was used to increase the stability of the MN. Each MN appeared tapered in shape, with a height of 600 microns and a bottom diameter of 300 microns (Fig. 2a, b). The patches were observed with a scanning electron microscope to display a uniform MN distribution and size, pointing to our successful preparation of the MNs (Fig. 2c, d).

The development of MNs is more conducive to targeted drug release and enhanced tissue absorption. Our study used cyclodextrin loaded tazarotene and exos to realize dual effects, and evaluated the drug release in vitro and exos release in vivo. On this basis, we assessed the degradation of MNs at different periods, noting that the surface morphology of MNs changed significantly with time. The MNs maintained their shape after degradation, even if they now harbored progressively more holes, which would also be favor of the exos and drugs releasing (Fig. 2e–j). Owing to the morphology of the gel, it has better degradability than the traditional MNs obtained after thorough drying, and their swelling during water absorption will also cause the pore size of the gel to become larger.

### Identification of HUVECs-exos

**Fig. 3:**
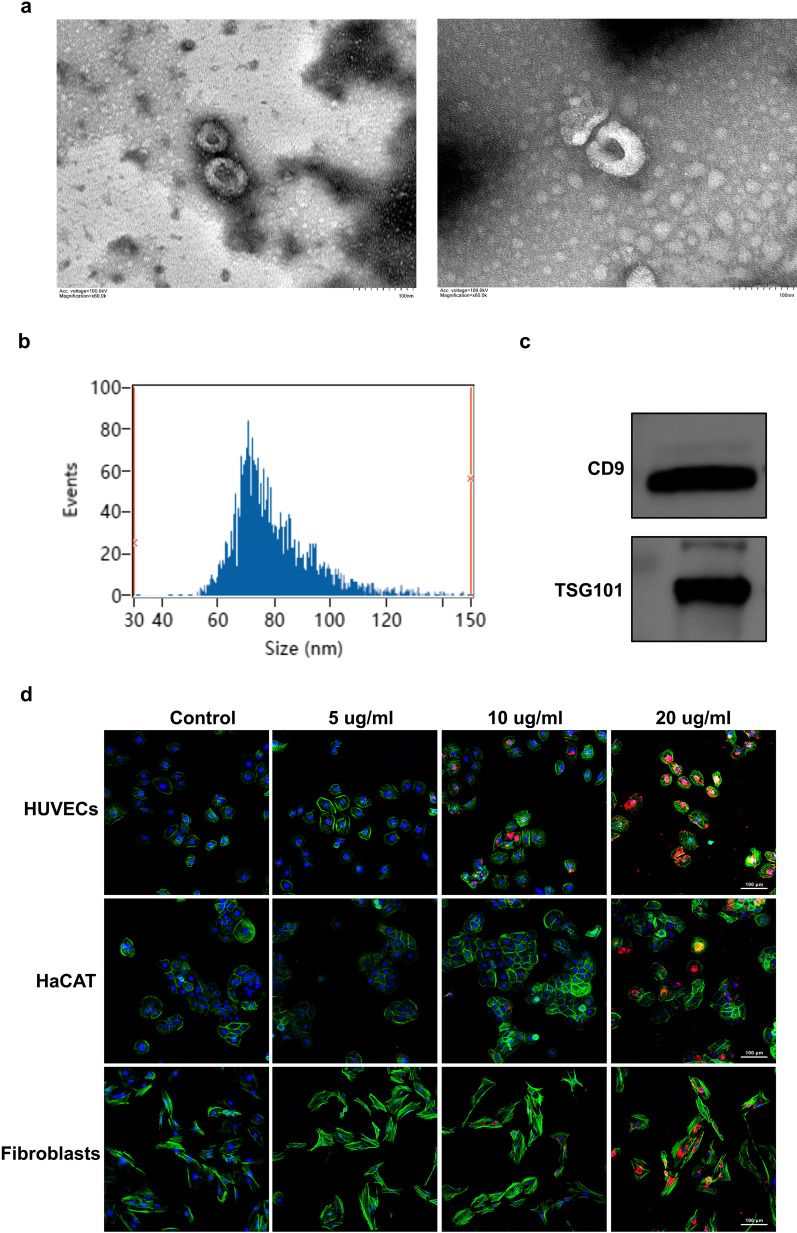
Characterization and internalization of HUVECs‐Exos. **a** Morphology of HUVECs-Exos observed by transmission electron microscopy. Scar bar, 100 nm. **b** Measurement of HUVECs-Exos population by NTA demonstrated a single‐peaked pattern (79.9 ± 0.1 nm in diameter). **c** Expression of exosome surface markers (CD9, TSG101) was confirmed by western blotting. **d** Confocal images of human foreskin fibroblasts, HaCAT, HUVECs, and incubated with PBS, 5 ug/ml, 10 ug/ml, 20 μg/ml PKH26-labeled HUVECs-Exos for 24 h. Scar bar, 100 μm

The identification of HUVECs-exos was achieved by TEM, NTA technology and Western blotting for the identification of TSG101 and CD9 markers of exos. TEM revealed that HUVECs-exos showed a cup‐shaped morphology (Fig. 3a). The size of HUVECs‐exos was measured by NTA, and the average size of HUVECs‐exos was 79.99 nm, which was in accordance with the previous study [36] (Fig. 3b). Western blot displayed that the HUVECs‐exos positively expressed surface markers such as CD9 and TSG101 (Fig. 3c). These findings demonstrated the successful isolation of HUVECs-exos.

### HUVECs-exos are internalized by HaCAT, HUVECs and fibroblasts

HaCAT, HUVECs and fibroblasts the three most essential cells in skin tissue were used to determine whether exos can be swallowed. After co-incubating of these three cells with PKH26-labeled exos for 24 h, the significant amounts of red fluorescent-labeled exos in the cytoplasm of these cells were observed, the uptake of HUVECs‐exos by these cells was dose-dependent and the red fluorescence intensity was the strongest when the concentration of the exos was 20 ug/ml, suggesting that HUVECs-exos could be internalized by these cells (Fig. 3d).

### Release of HUVECs-exos and Tazarotene by GelMA/PEGDA MNs

**Fig. 4:**
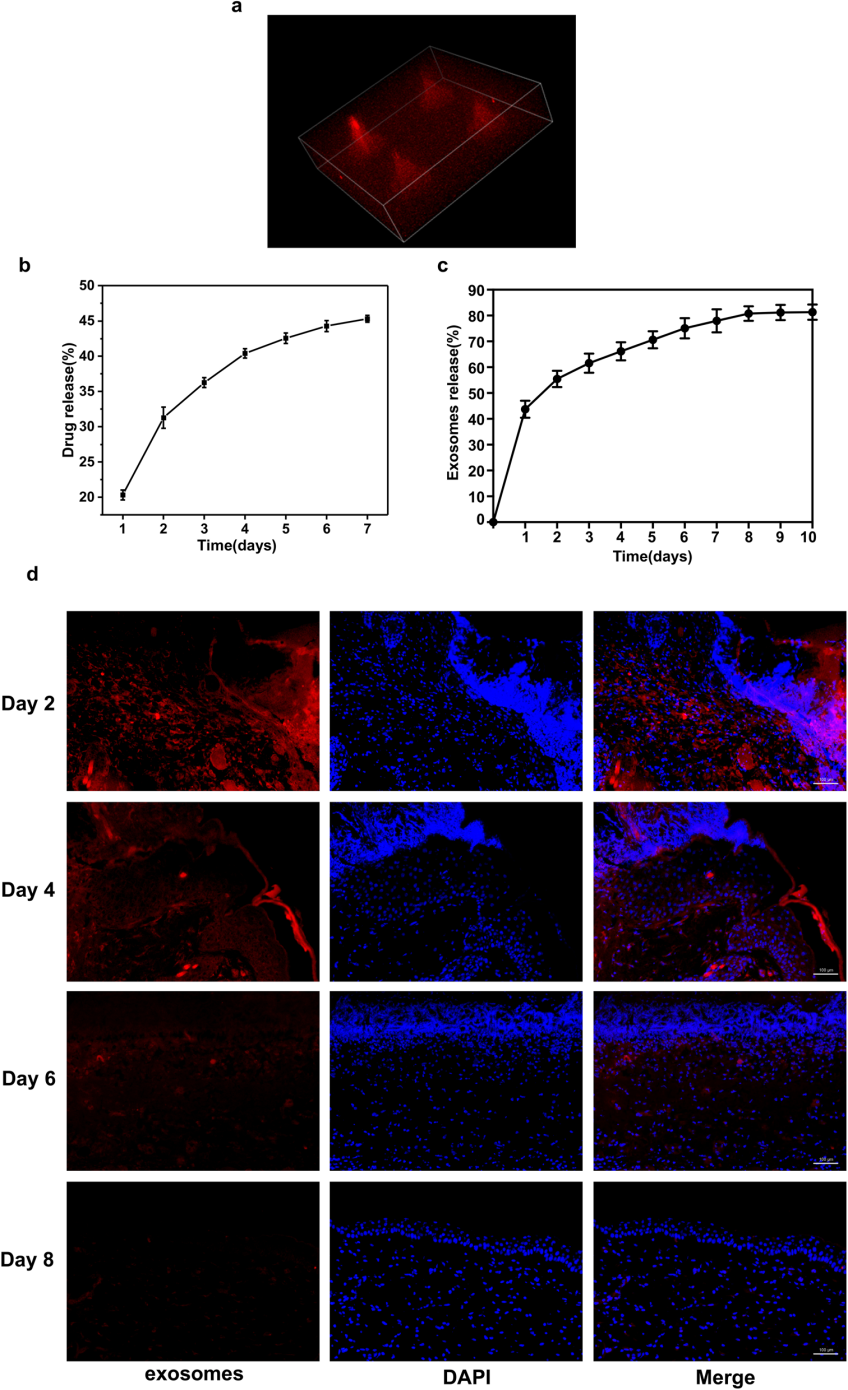
Release of HUVECs-Exos and Tazarotene by GelMA/PEGDA@T + exos MNs patch. **a** 3D reconstruction of confocal layer by layer scanning. **b** Tazarotene released curve of the GelMA/PEGDA@T + exos MNs patch. **c** Exosomes released curve of the GelMA/PEGDA@T + exos MNs patch. **d** Immunofluorescent staining of the skin covered with the GelMA/PEGDA@T + exos MNs patch at day 2, 4, 6, 8. Scar bar, 100 μm

Tazarotene had an obvious aboriginal peak at 351 nm. Due to the loading effect of cyclodextrin, drugs can be released slowly, and exos can also be steadily released from MNs. Our results showed that tazarotene release reached 20.3% on the first day and followed a gradual pattern at the later stage before reaching 45.1% at the end (Fig. 4b), indicating that MNs could effectively release drugs and could be used as a reference for drug release. An increasing number of exosomes proteins were detected by the Kit. The release curve was showed in Fig. 4c, it suggested that the MNs patch could realize sustained release of the exos in vitro, and reached over 80% cumulative release amount after 10 days.

PKH26-labeled exos were loaded into the GelMA/PEGDA MNs and observed under the confocal microscope. 3D reconstruction image revealed that the exos were distributed widely in the MNs (Fig. 4a, Additional file 1: Fig S4). The HUVECs-exos loaded GelMA/PEGDA MNs patches were covered to the shaved back skin of the diabetic C57BL mice. The skin was removed and then frozen section was performed. The sections were detected by fluorescence microscope. The results showed that exos were released gradually to the wound skin. The fluorescence intensity of the samples from day 0 to day 2, day 2 to day 4, day 4 to day 6, day 6 to day 8 was detected, and the fluorescence signal was still detectable on day 8 (Fig. 4d), suggesting that GelMA/PEGDA MNs patch could achieve continuous release of the exos in vivo.

### In vitro Biocompatibility of GelMA/PEGDA hydrogel

**Fig. 5:**
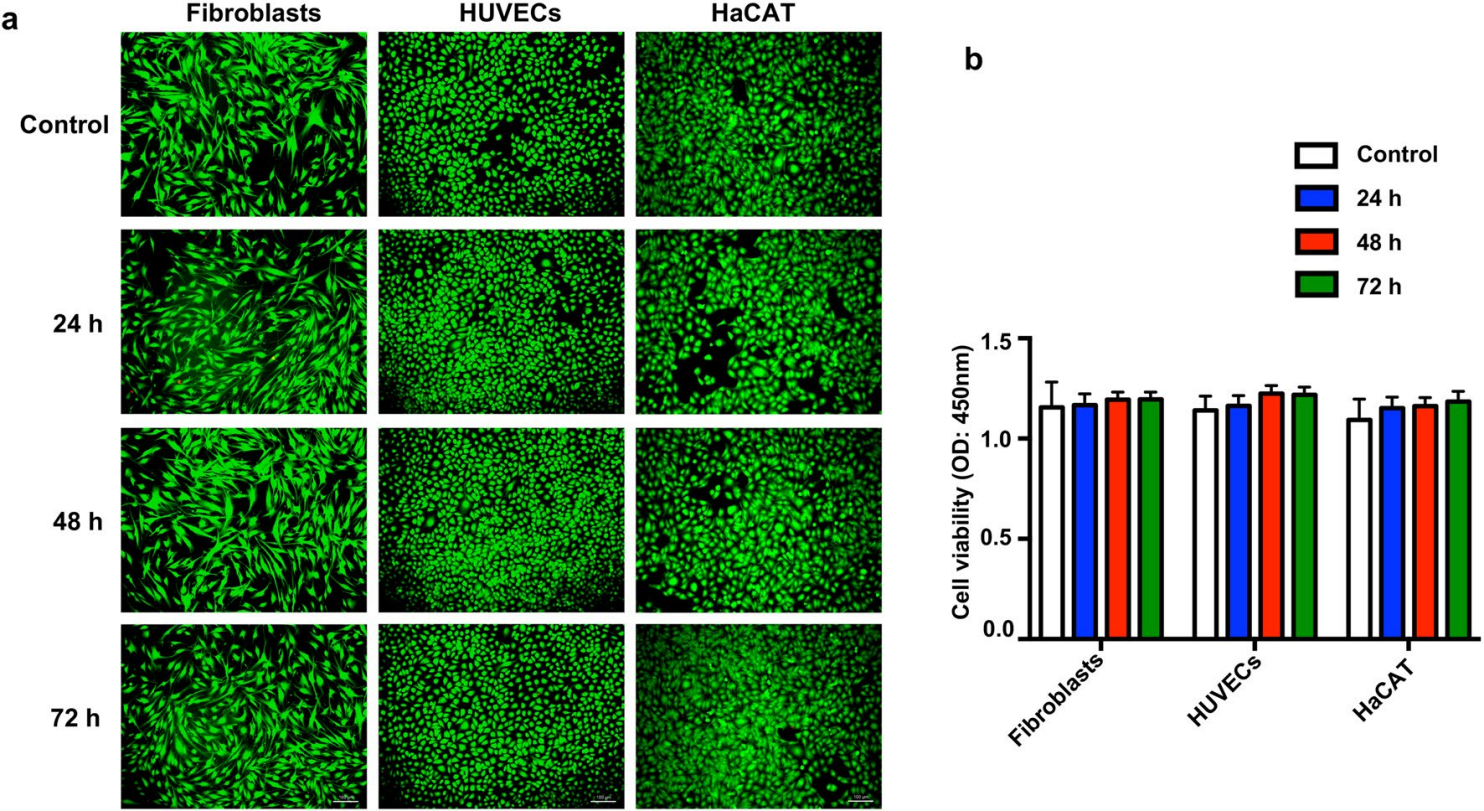
Biocompatibility of GelMA/PEGDA Hydrogel. **a** Survival ratio of fibroblasts, HUVECs, HaCAT cells treated with liquid extracts of GelMA/PEGDA hydrogel. Scar bar, 100 μm. **b** Cell viability was determined by CCK8 assay. *p < 0.05, **p < 0.01, ***p < 0.001, ****p < 0.0001

Biocompatibility is an important index for the evaluation of the quality of biomaterials. LIVE/DEAD cell staining assay and CCK-8 assay were used to test the cell viability and proliferation. As shown in Fig. 5a, b, the GelMA/PEGDA hydrogel ensured high cell viability for these cells. We also examined hydrogel degradation and found that the toxicity of the degradation products of hydrogel to cells was almost negligible (Additional file 1: Fig. S3).

### Influences on cell migration

**Fig. 6:**
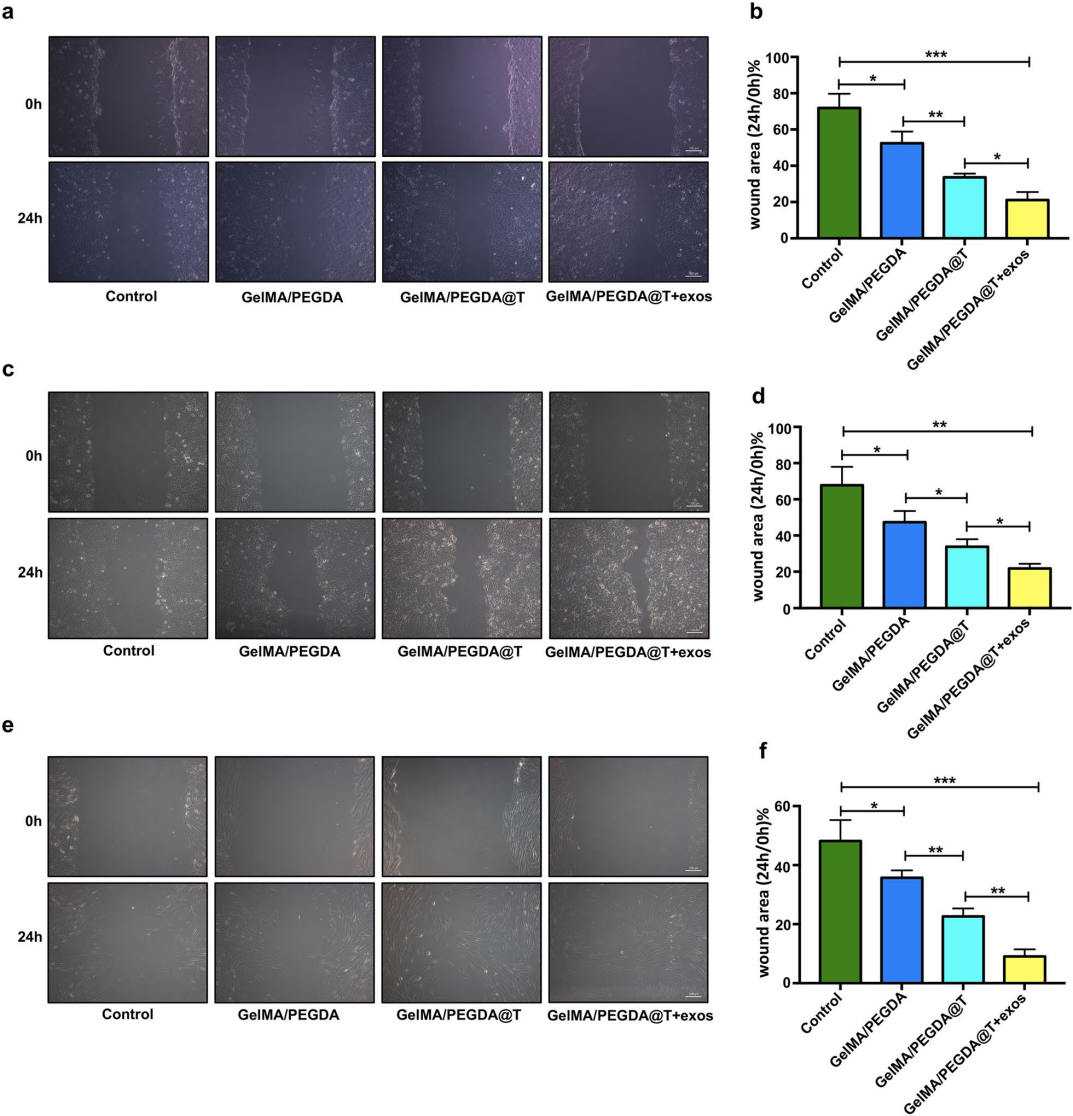
Representative images of the wound scratches of HUVECs **a**, HaCAT **c** and fibroblasts **e** with different treatment respectively. Scale bar: 100 μm. Statistical analysis of the healing area in the scratch assay of HUVECs **b**, HaCAT **d** and fibroblasts (**f**). *p < 0.05, **p < 0.01, ***p < 0.001, ****p < 0.0001

Cell migration is essential for wound healing, wound healing rate was estimated by using scratch assay in vitro. Keratinocytes, fibroblasts and endothelial cells, which are involved in the regeneration of wound healing. All cells were incubated in complete medium of 1% FBS in order to eliminate the promoting effect of serum on cell proliferation. Compared to the control group, the migration of GelMA/PEGDA MNs patch-treated fibroblasts, HaCAT, and HUVECs cells markedly improved (Fig. 6a–f). Furthermore, the GelMA/PEGDA@T + exos MNs patch group was significantly more potent than the GelMA/PEGDA MNs patch group (Fig. 6a–f), demonstrating that loading the HUVEC-exos and tazarotene to the MNs can synergistically increase the motility of these cells, which suggests that MNs can effectively control the release of exos and drugs to improve the migration function of the cells.

### Influences on angiogenesis

**Fig. 7:**
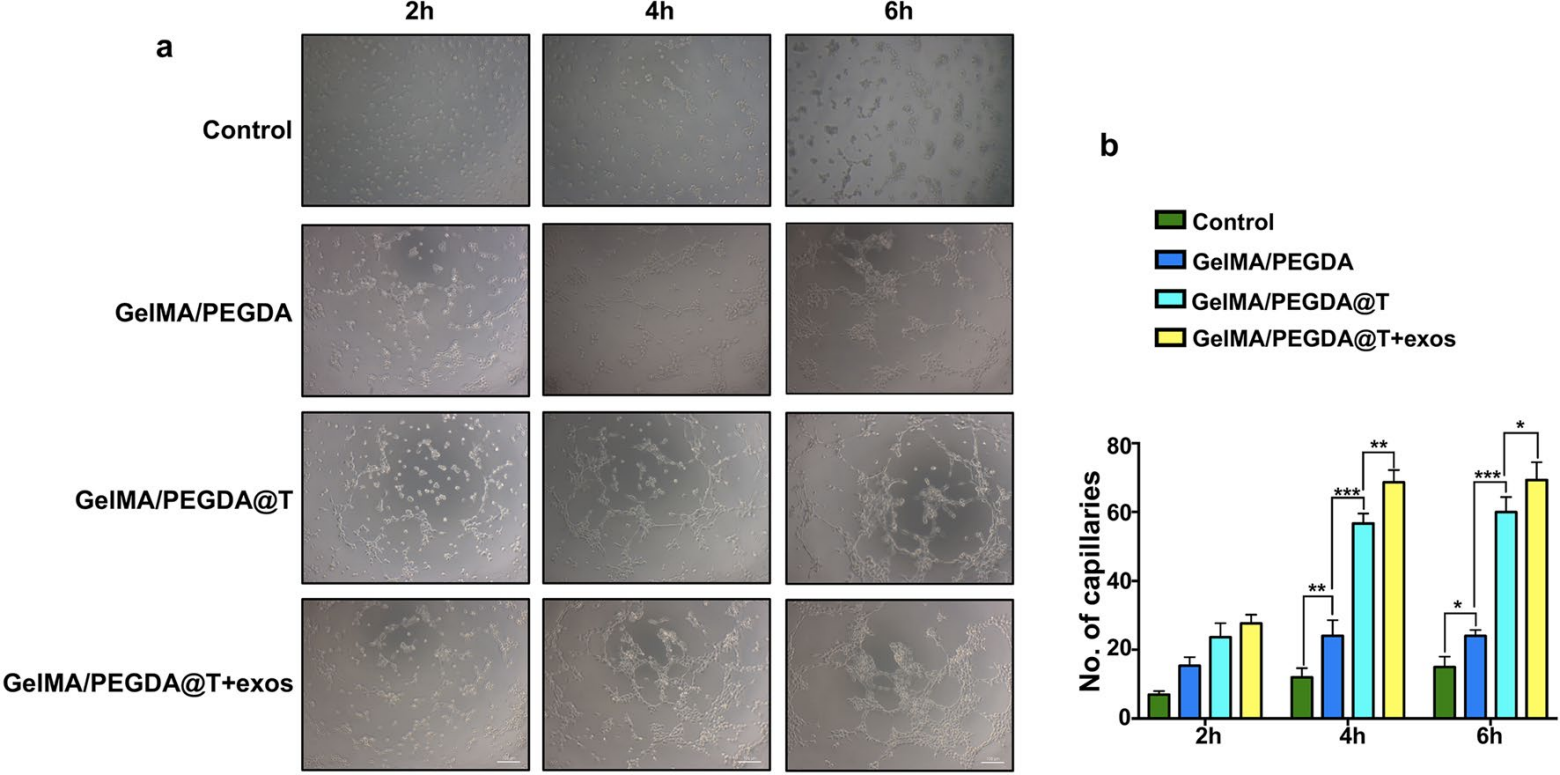
Microneedles induce angiogenesis. **a** Tube formation in HUVECs treated with different MNs patch compared with the control group at different time point. Scar bar, 100 μm. **b** Qualification of closed tubular structures. *p < 0.05, **p < 0.01, ***p < 0.001, ****p < 0.0001

Angiogenesis is one of the critical processes in wound healing, directly affecting the outcome of wound healing. Oxygen, nutrients and growth factors are delivered to injured sites through new blood vessels. Neovascularization is also essential to wound healing, because it starts from when the skin is injured to the end of wound remodeling. Vasculature assists in initial hemostasis, establishes a temporary wound matrix and reduces blood loss. As shown in Fig. 7a, b, closed tubular structures multiplied more in the GelMA/PEGDA@T MNs patch group than in the GelMA/PEGDA MNs patch group, demonstrating that the MNs can continuously release tazarotene in vitro, consistent with the results in Fig. 4b. MNs gradually released tazarotene, maintaining a high drug concentration in the medium. Although the angiogenic ability of HUVECs-exos has not been studied directly, we observed that blood vessels formed swifter in GelMA/PEGDA@T + exos MNs patch group than GelMA/PEGDA@T MNs patch group, proving that HUVECs-exos can stimulate angiogenesis. All results are based on MNs’ continuous release of biomolecules (Fig. 4b, c, d).

### In vivo wound healing experiments, Histologic and IF examination

**Fig. 8:**
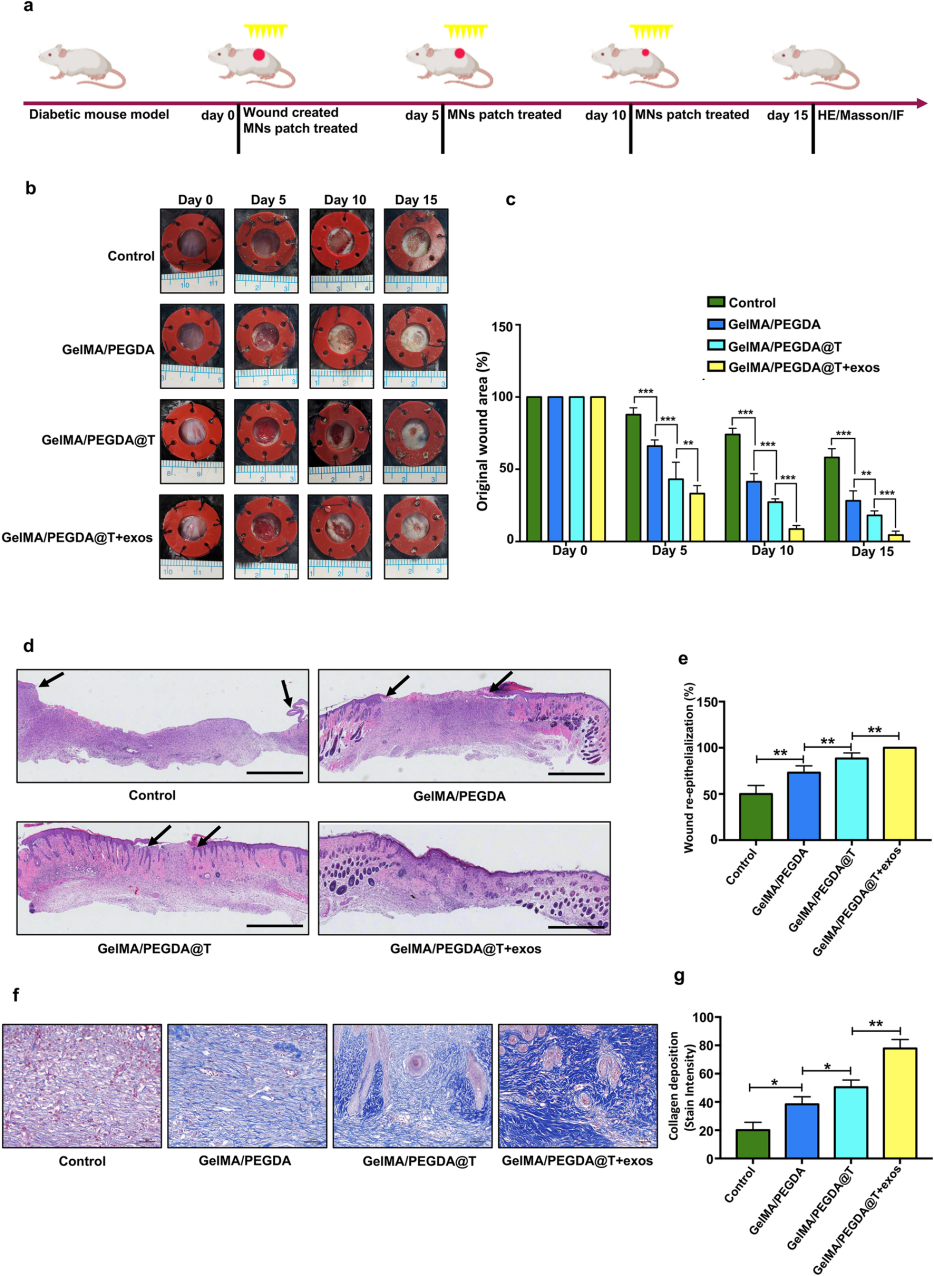
Effect of MNs patch on wound healing in a mouse model. **a** Schematic diagram of the diabetic wound model and treatment strategy. **b** Gross view of excisional wounds in mice treated with MNs patch at different time points. **c** Measurement of wound areas shown in b. **d** H&E staining analysis of wound sections following four different treatments at day 15 post-wounding. The single-headed arrows indicate the un-epithelialized areas. Scar bar = 1 mm. **e** Qualification of wound re-epithelialization shown in d. **f** Evaluation of collagen deposition by Masson staining at day 15 post-wounding. **g** Qualification of the stain intensity of blue collagen shown in f. Scar bar = 100 μm. *p < 0.05, **p < 0.01, ***p < 0.001, ****p < 0.0001

To verify our hypothesis that the GelMA/PEGDA@T + exos MNs patch promotes diabetic wound healing, full-thickness wounds on the dorsum of the C57BL mice were established. Blank group, GelMA/PEGDA MNs patch group, GelMA/PEGDA@T MNs patch group were used as controls to support comparison. Wound area (%) was defined as: residual wound area at day ‘X’) / Wound area at day ‘0’ × 100%. As shown in Fig. 8b, c, the wound closure rate in GelMA/PEGDA@T MNs patch group was significantly higher than control group and GelMA/PEGDA MNs patch group at days 10 and 15 post-wounding. The GelMA/PEGDA@T + exos MNs patch group’s wound healing effect was superior to that of the GelMA/PEGDA@T MNs patch group due to the cell migration and angiogenesis-promoting property of the HUVECs-exos. We further evaluated the tensile stress of the back skin of mice to reflect the repair effect after 15 days of treatment (Additional file 1: Fig. S5). The skins of mice in the GelMA/PEGDA@T + exos MNs patch group displayed the most robust tensile strength after repair, which also reflected the best repair effect. Re-epithelialization and wound contraction are the main approaches to skin wound healing. For example, rodents heal primarily through contraction, while re-epithelialization in humans accounts for 70–80% of wound repair, this process relies on the accelerating migration of keratinocytes. H&E staining showed GelMA/PEGDA@T MNs patch group and GelMA/PEGDA@T + exos MNs patch group had a higher wound healing rates than the GelMA/PEGDA MNs patch group, indicating better re-epithelialization properties by the former two (Fig. 8d, e). During the healing process of skin injuries, collagen deposition and remodeling are conducive to tissue repair and regeneration, with collagen fibers forming the dermal structure callus providing tension to the skin and scar tissue. We further confirmed the collagen deposition using Masson staining, and established that collagen remodeling in the GelMA/PEGDA@T + exos MNs patch group and GelMA/PEGDA@T MNs patch group was superior to that in the other groups (Fig. 8f, g).

**Fig. 9:**
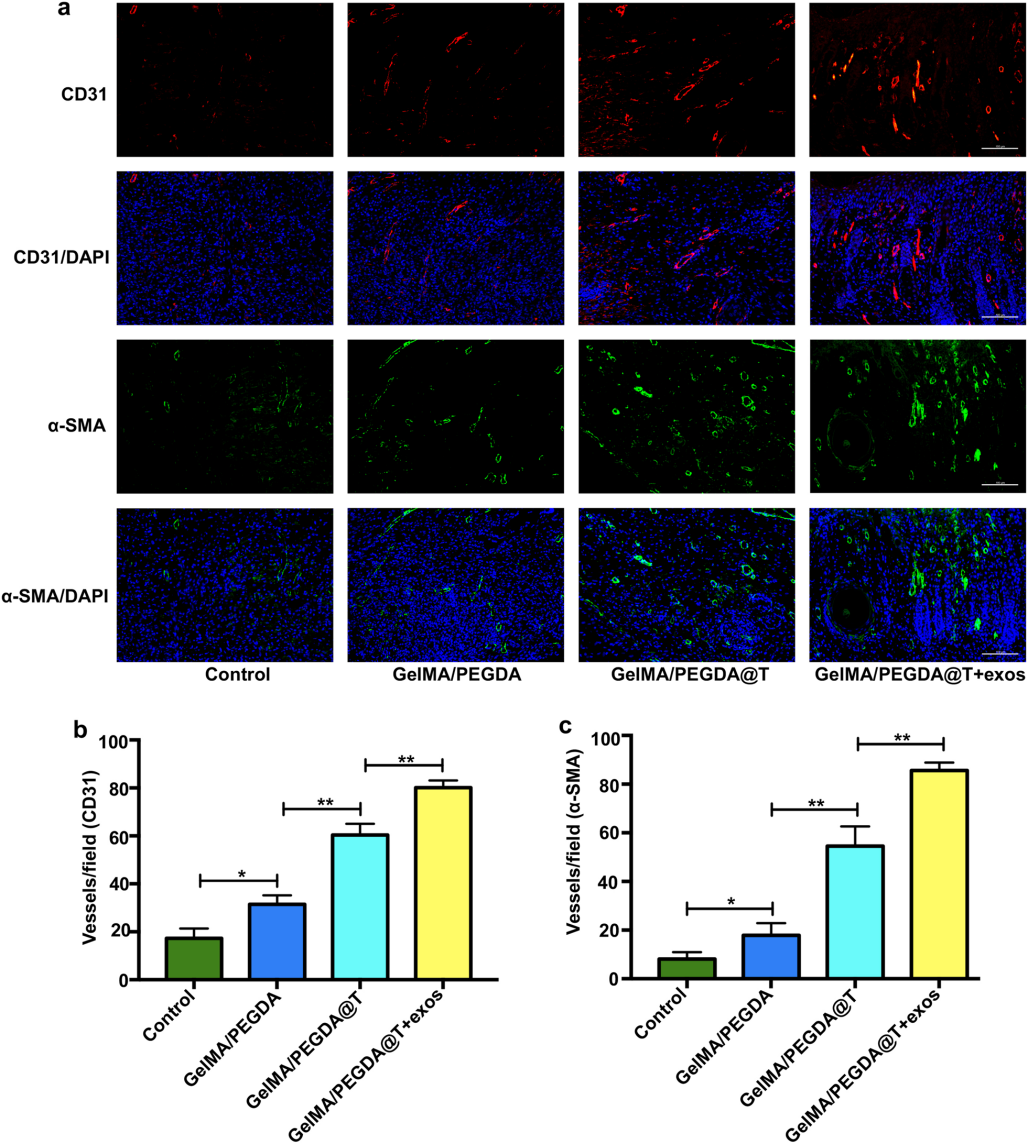
Immunofluorescent staining of wound sections given the above treatments at day 15 post-wounding. **a** Endothelial cells (CD31), smooth muscle cells (a-SMA), and cell nuclei (DAPI) were stained with red, green, and blue colors. **b**, **c** Qualification of the stain intensity of CD31 and a-SMA shown in a. *p < 0.05, **p < 0.01, ***p < 0.001, ****p < 0.0001

Reduced angiogenesis in diabetic wound bed are considered critical to the slow healing of the skin. Endothelial cells migrate to the damaged area to form new blood vessels, carrying oxygen and nutrients to the wound site to support angiogenesis. Reportedly, tazarotene promotes vascular regeneration. To further scrutinize the angiogenesis in these MNs patch-treated mice, immunofluorescence staining analysis of CD31 and α-SMA was conducted to assess the angiogenesis near the wound areas. Positively stained vessels of CD31 and α-SMA in the GelMA/PEGDA@T MNs patch group were considerably higher than in the GelMA/PEGDA MNs patch group at day 15 (Fig. 9a–c), signifying that the MNs can continuously release tazarotene and promote angiogenesis around the wound surface. Additionally, the relative densities of CD31 and α-SMA were higher in the GelMA/PEGDA@T + exos MNs patch group than in the other three groups (Fig. 9a–c), HUVECs-exos loaded into the MNs further induced increased angiogenesis on the basis of GelMA/PEGDA@T MNs patch treatment.

Substantial differences were noted in collagen deposition, re-epithelialization and enhanced CD31 and α-SMA expression between control group and GelMA/PEGDA MNs patch group on 15 day (Fig. 9a–c). The results also proved that the structure and properties of GelMA/PEGDA MNs were not affected by tazarotene and HUVECs-exos loading. GelMA/PEGDA MNs patch carried tazarotene and HUVECs-exos, continuously releasing them to the wound site during the repair process, promoting vascular regeneration and accelerating skin repair. Therefore, the newly synthesized GelMA/PEGDA@T + exos MNs patch is an excellent active molecule and drug carrier for diabetic wound healing.

## Discussion

The incidence of diabetes is increasing, and the survival rate of diabetes-related amputations is remains nonideal [37]. DFU patients face colossal treatment costs and severely reduced quality of life [38, 39]. Therefore, developing a new dressing conducive to the healing of diabetic ulcers is of significant necessity and benefit to both patients and society. The clinical treatment of diabetic wounds primarily focuses on anti-infection and local artificial dressings [40]. Our main objective in this research was to evaluate the capacity of the methacrylate gelatin MNs patch to support the long-term release of HUVECs-exos and tazarotene to promote diabetic wound repair.

MNs have more prominent advantages over traditional drug delivery systems [41]. While the latter approach ensures that drugs work faster through the skin and the spray method has a broader coating area, these systems, nevertheless, either inevitably result in the loss of substantial amounts of drugs or the drug cannot ensure long-term delivery. This dilemma has seen hydrogel administration which provides the moist environment required in wound repair processes and guarantees the long-term timeliness of administration and better coverage of wounds attract extensive attention in recent years. Because traditional hydrogel cannot penetrate the skin, which mitigates the therapeutic effect of drugs, delivery systems capable of penetrating the skin and providing better absorption of the drugs, must be created. Based on this, MNs, which can penetrate the skin via injection and not cause significant pain, have emerged as the most suitable delivery system. At the same time, MNs also possess characteristics common to traditional hydrogels and are suitable for the treatment of refractory wounds.

This study used GelMA and PEGDA as matrix materials and add β-CD-AOI_2_ for tazarotene delivery. The incorporated exos are released into the tissue through MNs, and the transcutaneous delivery efficiency of tazarotene was improved. We first analyzed the material’s properties, using infrared and nuclear magnetic methods to establish the successful preparation of GelMA and β-CD-AOI_2_ [30]. Next, we examined the impact of PEGDA on the properties of hydrogels, revealing that the addition of PEGDA amplified the cross-linking density and further strengthened the hydrogels, which is of remarkable significance to the preparation of MNs. Before preparing MNs, we concentrated the pre-solution, which is a necessary step, and evaluated the changes in the morphology of the hydrogel pre- and post-concentration. We found that the pore size of the hydrogel was significantly smaller due to the increase in the amount of the pre-solution post concentration, and the increase in the cross-linking density. Based on the above research and referring to the previous MNs preparation methods, we used the mold method, a commonly used MNs preparation method, to prepare MNs [42, 43]. Through scanning electron microscopy, we found that the obtained MNs had excellent morphology for the effectively penetration of the skin and the steady release of drugs and exos for diabetic skin repair. We further established that MNs have remarkable degradation properties and can accelerate the release of drugs and exos, which is beneficial to the later repair of wounds.

Three steps include inflammation, new tissue formation, and remodeling were involved in the process of wound repair. The proliferation, migration and angiogenesis of keratinocytes, fibroblasts, and endothelial cells are characteristics of the second step [44, 45]. Therefore, we evaluated the function of different MNs on these three cells mentioned above and observed that the GelMA/PEGDA MNs patch group cells grew faster and had slightly better epithelialization than cells in the control group thanks to the pore structure of the hydrogel itself, which is conducive to cell migration. In addition, the GelMA/PEGDA@T MNs patch group has been shown to have significant angiogenic capacity, both in vitro and in vivo angiogenesis experiments. This study demonstrated that our MNs patch provided a universally intelligent drug delivery system that minimizes tazarotene’s low water solubility deficiency to promote angiogenesis. The MN has a larger surface area than traditional dressings, facilitating efficient drug delivery. Angiogenesis is particularly crucial to the proliferation phase of wound healing [46, 47], while endothelial cells are responsible for forming new vessels [48–50]. However, research on endothelial cell derived exos is scant. This investigation loaded HUVECs-exos into GelMA/PEGDA@T MNs and examined their therapeutic effects in a diabetic mouse model. Adding HUVECs-exos to the GelMA/PEGDA@T MNs patch group, resulted in a more significant promotion of wound healing associated with collagen deposition, epithelial regeneration, and angiogenesis, demonstrating that GelMA/PEGDA MNs patches could realize effective delivery of the biomolecules.

## Conclusion

We have developed a controllable biomolecules released MNs patch comprising GelMA/PEGDA hydrogel for promoting wound healing. These GelMA/PEGDA MNs can encapsulate tazarotene and HUVECs-exos to release them around the wound site, drastically accelerating collagen deposition, epithelial regeneration and angiogenesis, indicating that tazarotene-HUVECs-exos loaded GelMA/PEGDA MNs patch is a promising clinical treatment approach for DFU.

## Supplementary Information

**Additional file 1: Figure S1.** Compressive behavior of GelMA/PEGDA hydrogel. **Figure S2.** SEM images of hydrogel after concentrated. Scale bar = 200 μm (**a**, **b**, **c**, **g**, **h**, **i**), scale bar = 100 μm (**d**, **e**, **f**, **j**, **k**, **l**). **Figure S3.** Biocompatibility of the degradation products of GelMA/PEGDA hydrogel. **a** Survival ratio of fibroblasts, HUVECs, HaCAT treated with the degradation products of GelMA/PEGDA hydrogel after 7, 14 days, scale bar: 100 μm. **b** Cell viability was determined by CCK8 assay. *p < 0.05, **p < 0.01, ***p < 0.001, ****p < 0.0001. **Figure S4.** HUVECs-Exos were loaded into MNs. **a**, **b** Planar imaging of the Dil-labeled GelMA/PEGDA@T+exo MNs. Scar bar in **a**, 1000 μm. Scar bar in **b**, 100 μm. **c** 3D reconstruction of confocal layer by layer scanning. **Figure S5.** Tensile stress of the back skin of mice at 15 post-wounding. **a** Tensile force detection device. **b** Tensile force curve of the four groups of the mice.

## Data Availability

All data generated and analyzed during this research are included in this published article.
