# Supplementary material for: GelMA/PEGDA microneedles patch loaded with HUVECs-derived exosomes and Tazarotene promote diabetic wound healing
Source: J Nanobiotechnology. 2022 Mar 19;20:147. doi: 10.1186/s12951-022-01354-4 (PMC8934449; doi:10.1186/s12951-022-01354-4)
Supplement: Supplementary file 1 — Additional file 1: Figure S1. Compressive behavior of GelMA/PEGDA hydrogel. Figure S2. SEM images of hydrogel after concentrated. Scale bar = 200 μm (a, b, c, g, h, i), scale bar = 100 μm (d, e, f, j, k, l). Figure S3. Biocompatibility of the degradation products of GelMA/PEGDA hydrogel. a Survival ratio of fibroblasts, HUVECs, HaCAT treated with the degradation products of GelMA/PEGDA hydrogel after 7, 14 days, scale bar: 100 μm. b Cell viability was determined by CCK8 assay. *p < 0.05, **p < 0.01, ***p < 0.001, ****p < 0.0001. Figure S4. HUVECs-Exos were loaded into MNs. a, b Planar imaging of the Dil-labeled GelMA/PEGDA@T+exo MNs. Scar bar in a, 1000 μm. Scar bar in b, 100 μm. c 3D reconstruction of confocal layer by layer scanning. Figure S5. Tensile stress of the back skin of mice at 15 post-wounding. a Tensile force detection device. b Tensile force curve of the four groups of the mice. [file 12951_2022_1354_MOESM1_ESM.docx]

**GelMA/PEGDA microneedles patch loaded with HUVECs-derived exosomes and** **Tazarotene promote diabetic wound healing**

Meng Yuana, 1, Kun Liu b, 1, Tao Jiang a, Shengbo Li a, Jing Chen a, Zihan Wu a, Wenqing Li c, Rongzhi Tan c, Xiaofan Yang a *, Honglian Dai b *, Zhenbing Chen a *

a Department of Hand Surgery, Union Hospital, Tongji Medical College, Huazhong University of Science and Technology, Wuhan 430022, China.

b State Key Laboratory of Advanced Technology for Materials Synthesis and Processing, Wuhan University of Technology, Wuhan 430070, China.

c Department of Hand and Foot Surgery, Huazhong University of Science and Technology Union Shenzhen Hospital, Shenzhen 518052, China.

**Corresponding Authors**

Zhenbing Chen, Department of Hand Surgery, Union Hospital, Tongji Medical College, Huazhong University of Science and Technology, Wuhan 430022, China.

E-mail addresses: zhenbing_chen@163.com

Honglian Dai, State Key Laboratory of Advanced Technology for Materials Synthesis and Processing, Wuhan University of Technology, Wuhan 430070, China.

E-mail addresses: daihonglian@whut.edu.cn

Xiaofan Yang, Department of Hand Surgery, Union Hospital, Tongji Medical College, Huazhong University of Science and Technology, Wuhan 430022, China.

E-mail addresses: 2017xh0119@hust.edu.cn

1Meng Yuan and Kun Liu contributed equally to this work.

**
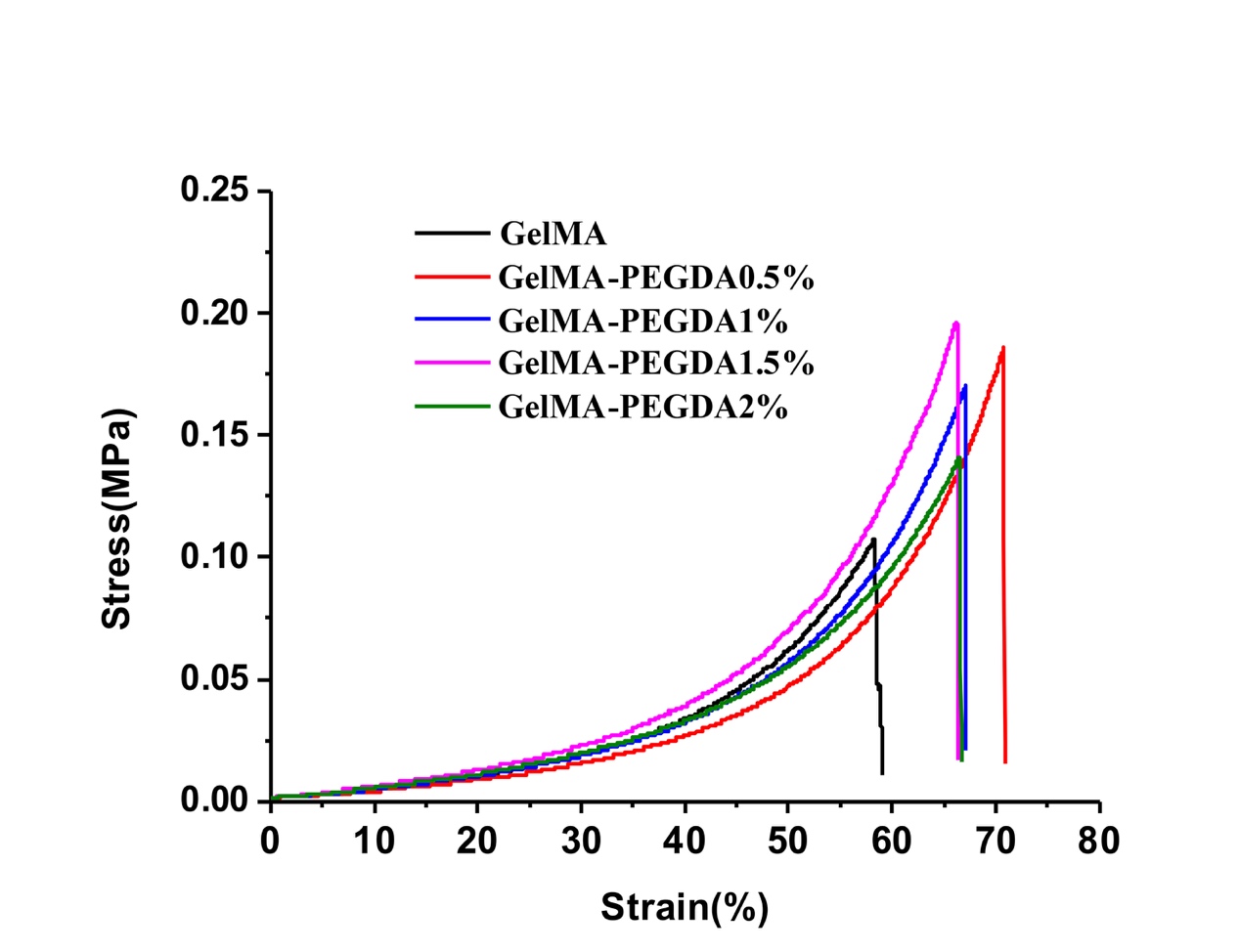
**

**Figure s1.** Compressive behavior of GelMA/PEGDA hydrogel.


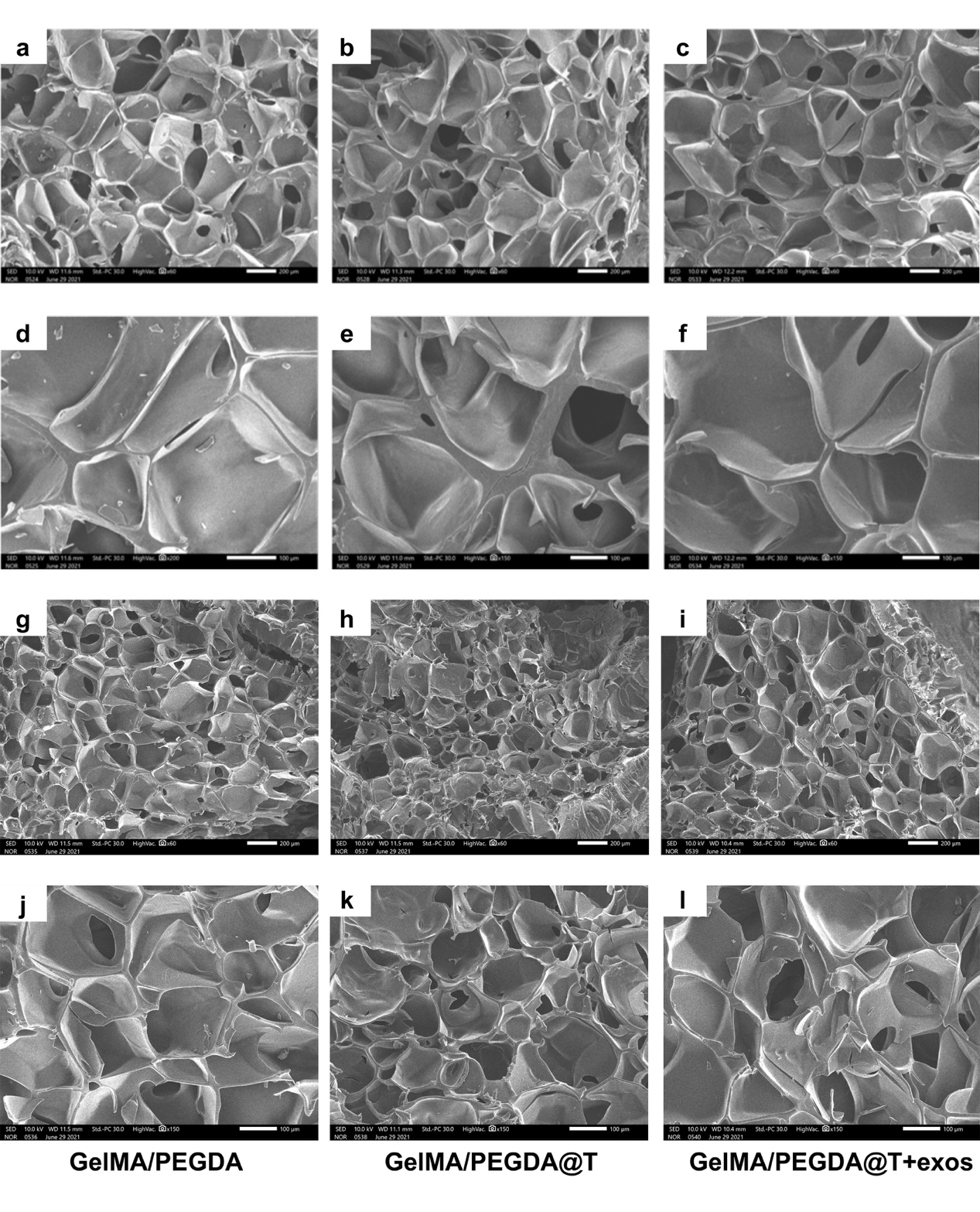


**Figure s2.** SEM images of hydrogel after concentrated. scale bar=200 μm (a, b, c, g, h, i), scale bar=100 μm (d, e, f, j, k, l).


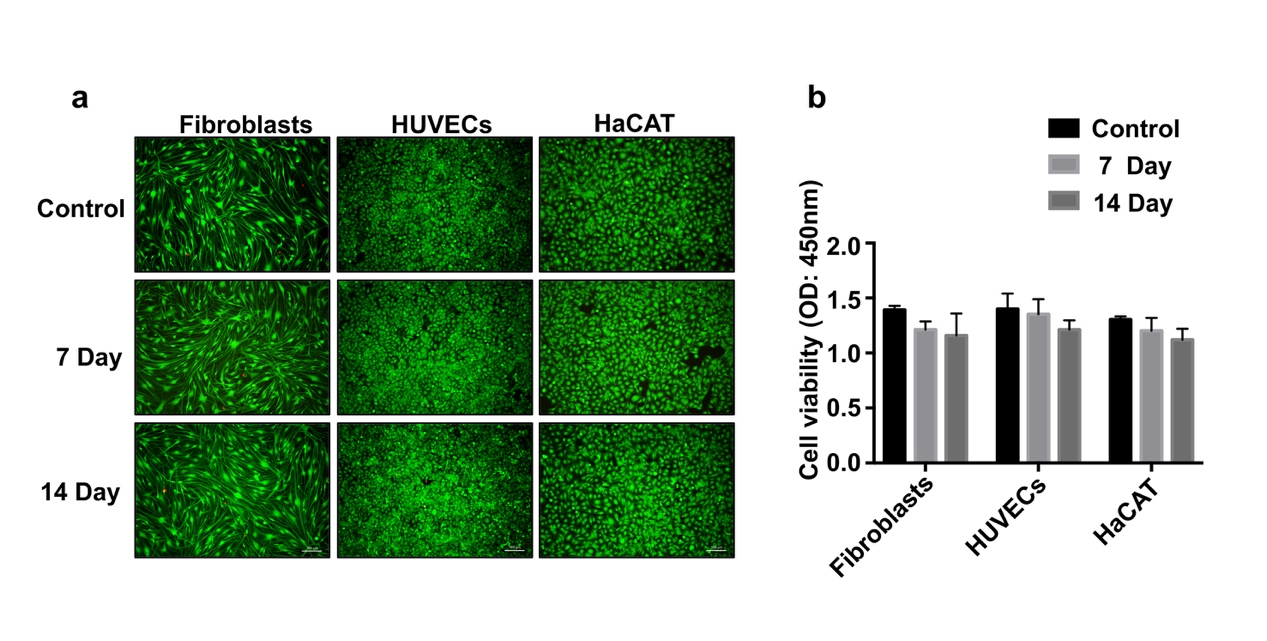


**Figure s3**. Biocompatibility of the degradation products of GelMA/PEGDA hydrogel. (a) Survival ratio of fibroblasts, HUVECs, HaCAT treated with the degradation products of GelMA/PEGDA hydrogel after 7, 14 days, scale bar: 100 μm. (b) Cell viability was determined by CCK8 assay. *p < 0.05, **p < 0.01, ***p < 0.001, ****p <0.0001


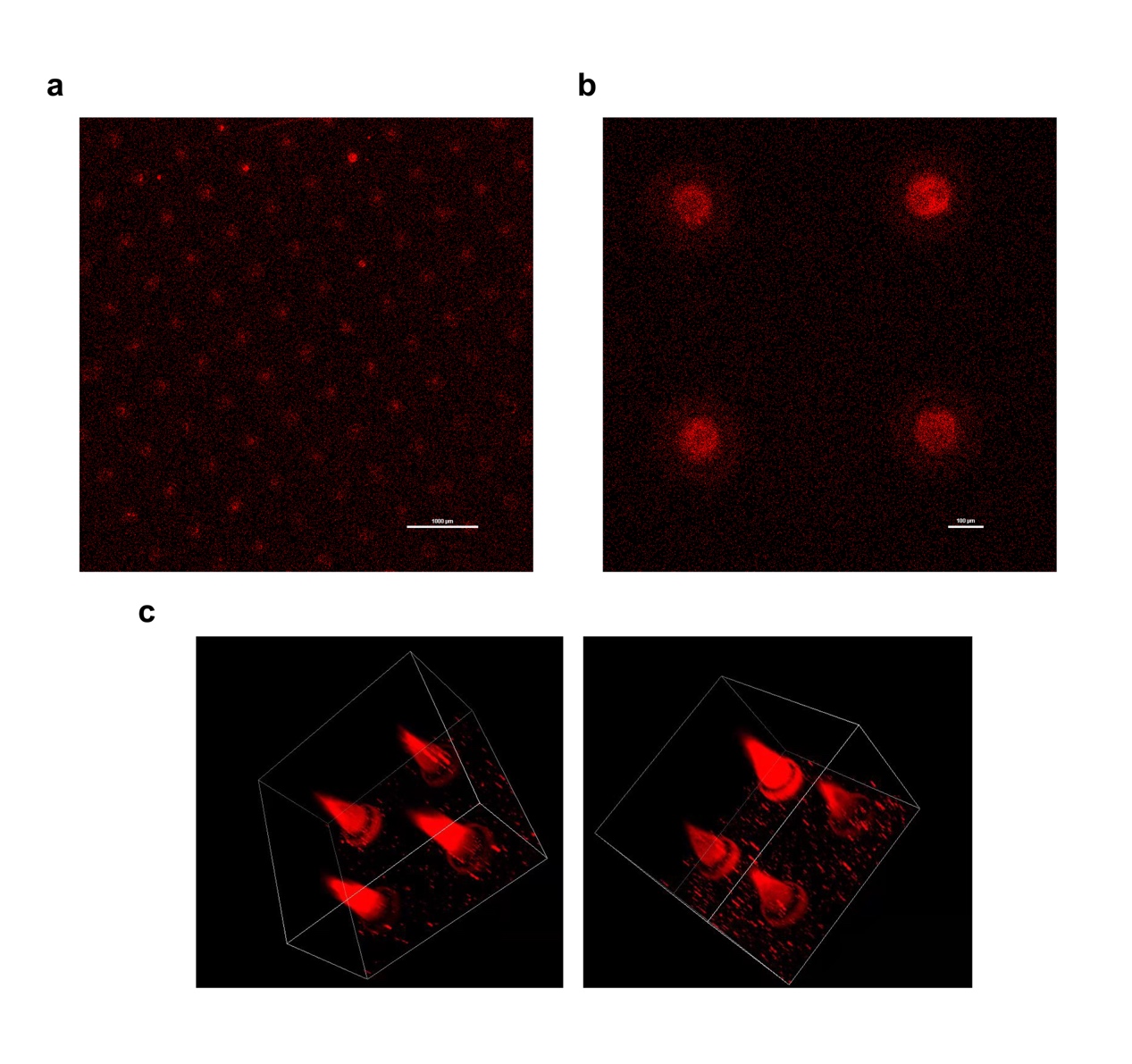


**Figure s4.** HUVECs-Exos were loaded into MNs. (a, b) Planar imaging of the Dil-labeled GelMA/PEGDA@T+exo MNs. Scar bar in a, 1000 μm. Scar bar in b, 100 μm. (c) 3D reconstruction of confocal layer by layer scanning.


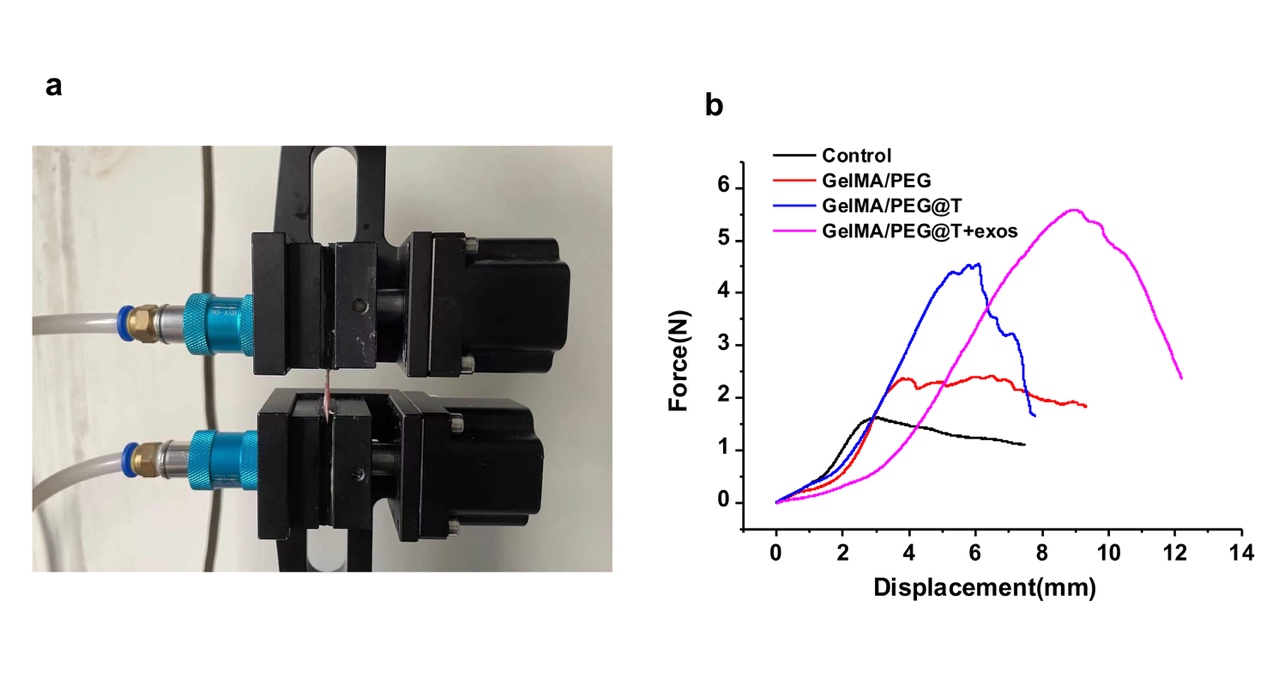


**Figure s5.** Tensile stress of the back skin of mice at 15 post-wounding. (a) Tensile force detection device. (b) Tensile force curve of the four groups of the mice.
